# Patient experience of whole body diffusion weighted magnetic resonance imaging (WB-MRI) for staging myeloma

**DOI:** 10.1186/1470-7330-15-S1-P13

**Published:** 2015-10-02

**Authors:** S Otero, M Kaiser, C Pawlyn, S Giles, E Scurr, C Messiou

**Affiliations:** 1 The Royal Marsden Hospital (Surrey), Sutton, Surrey, SM2 5PT, UK

## Aim

WB-MRI provides a fast, highly sensitive assessment of disease burden in myeloma. The 2015 International Myeloma Working Group consensus statement recommends WB-MRI for staging asymptomatic myeloma and for workup of solitary bone plasmacytoma. The technique is noisy, employs whole body surface coils, and takes longer than a standard MRI spine. We assessed patient experience of WB-MRI and identified causes for incomplete examinations.

## Methods

36 consecutive patients undergoing WB-MRI for myeloma (whole body DWI and Dixon, fast T1w and T2w spine) were included. Patients anonymously completed a ten-question survey about their experience. The reporting radiologist recorded technical details and radiological findings.

## Results

WB-MRI was well tolerated in most patients. 89% completed the protocol; kyphosis and claustrophobia were causes of incomplete studies. 85% found the scan ‘*not at all unpleasant*’ or ‘*not too unpleasant*’.

96% were satisfied with the quality of information provided to them prior to the examination. 93% had had a previous MRI and 86% were not worried about having WB-MRI. 93% would have a repeat study.

Average scan length was 48 minutes. Two-thirds of patients found this acceptable. 18% of patients cited claustrophobia as the reason for finding the examination too long. 44% of patients had chronic vertebral fractures, but this did not correlate with the level of discomfort experienced. Half of scans were positive for active disease.

## Conclusions

Patients report a high level of satisfaction with WB-MRI at our institution. The protocol was completed in almost all patients, and most stated they would have a repeat study.

